# Dataset of IoT-based energy and environmental parameters in a smart building infrastructure

**DOI:** 10.1016/j.dib.2024.110769

**Published:** 2024-07-29

**Authors:** Adel Oulefki, Abbes Amira, Fatih Kurugollu, Bassel Soudan

**Affiliations:** Smart Sustainable Cities Research Group (S2C), University of Sharjah, United Arab Emirates

**Keywords:** Internet of Things (IoT), Smart buildings, Energy efficiency, Environmental sensing, Occupancy analytics, Power consumption metrics, Sustainable infrastructure, Data analysis

## Abstract

This data article presents a detailed dataset collected as part of the University of Sharjah's (UoS) strategic initiative towards transforming into a smart campus by 2030. Collected from January 1st, 2024, to June 20nd, 2024, from key facilities including offices, labs, and communal spaces, the dataset encompasses precise energy consumption metrics and environmental conditions monitored via Internet of Things (IoT) sensors. It features appliance-specific power data (watts, voltage, kWh) alongside environmental parameters such as temperature, humidity, and occupancy rates. Distinctively, this dataset includes Markov Transition Field (MTF) visualizations, converting time series data into analytical 2D images, which facilitates advanced data interpretations suitable for Deep and transfer learning applications. Aimed at supporting research in energy management and intelligent system development, this dataset offers comprehensive insights into the operational dynamics of a transitioning smart campus, providing both raw and processed forms of data to accommodate diverse research needs.

Specifications Table

**Table:** 

Subject	Environmental Science; Information Systems and Technology; Energy Efficiency.
Specific subject area	IoT data on energy consumption and environmental conditions in smart buildings, augmented with Digital Twin (DT) for enhanced simulation and analysis.
Type of data	Type of data: Tables, Figures Data format: CSV (for sensor readings), PNG (for MTF images)
Data collection	Data were collected using IoT sensors (energy consumption meters, temperature, humidity, and occupancy sensors) deployed throughout a mixed-use building that accommodates offices, meeting rooms, a kitchen, a mailroom, research labs, and lecture halls. Energy data were recorded from devices like coffee machines, microwaves, etc., and environmental data were captured in key areas (Interdisciplinary lab, kitchen, and mailroom). Data were processed and visualized using MTF for pattern analysis
Data source location	Data was gathered at the University of Sharjah (UoS), with the Computer Science Department overseeing data storage and management. The sensors were strategically placed in specific locations, including the Interdisciplinary Laboratory, kitchen, and mailroom, to ensure comprehensive data collection. **Location of Data Collection**: University of Sharjah - University City Rd - University City - Sharjah https://maps.app.goo.gl/ZkKLS22bu5C3rkbp8
Data accessibility	**Repository name**: Zenodo Data identification number (or DOI or persistent identifier): 10.5281/zenodo.12750891 **Direct URL to data**: https://doi.org/10.5281/zenodo.12750891 **Instructions for accessing these data**: The data are openly accessible and can be downloaded directly from the provided URL. No special permissions or credentials are required for access.

## 1 Value of the Data

• The dataset provides an in-depth look at energy consumption and environmental parameters in a campus setting, making it an invaluable resource for researchers interested in energy efficiency, IoT applications in smart buildings, and environmental monitoring. By offering detailed, appliance-level energy usage alongside environmental conditions, the data enable a multifaceted analysis of building operations.
• Researchers and the community can utilize this dataset to benchmark energy consumption patterns, develop predictive models for energy usage, or enhance algorithms for intelligent building management systems. The inclusion of time-stamped, appliance-specific data allows for detailed temporal analysis and can help in identifying trends and potential areas for energy-saving interventions.
• The data serves as a foundational tool for studies in energy conservation strategies, providing a basis for comparing the effectiveness of different energy-saving measures within campus buildings. By analyzing this dataset, researchers can contribute to the development of more sustainable and energy-efficient building practices.
• This dataset can also support educational purposes, offering practical data for students and academics in fields comparable to environmental science, engineering, and data science to analyze and interpret real-world IoT data, fostering a deeper understanding of the interplay between technology and sustainability.
• The comprehensive nature of the dataset, covering various devices and environmental conditions, allows for interdisciplinary research opportunities, where scientists from different fields can collaborate to explore new insights into smart building ecosystems, IoT device performance, and the relationship between human occupancy patterns and energy usage.

## 2 Background

The compilation of this dataset was motivated by the evolving necessity in the energy efficiency sector to develop sophisticated computational models that accurately analyse energy behaviour patterns within campus buildings. Similar to how detailed appliance-level and environmental data have transformed energy efficiency modelling in residential contexts, there exists a parallel demand in educational campus environments to leverage such data to craft advanced Artificial Intelligence (AI)-based solutions [1,2].

The advent of AI in building energy management, particularly through anomaly detection systems, represents a significant stride towards optimizing energy usage and detecting inefficiencies. As highlighted in recent research, these AI-driven systems are pivotal in identifying deviations from normal energy consumption patterns, thereby facilitating timely interventions to curb waste and enhance operational efficiency [3]. By integrating AI to systematically analyse and react to energy data, institutions can significantly reduce operational costs and support sustainable energy practices.

Our dataset is meticulously designed to address these needs, offering an exhaustive collection of detailed energy consumption data along with environmental conditions measured within a university setting [4]. It aims to assist in the creation and refinement of machine learning models focused on advancing energy classification, anomaly detection, and the development of recommendation systems. This initiative aligns with the emerging trends where AI applications in energy management not only emphasize cost reduction and environmental stewardship but also bolster the operational efficiency of building management systems.

In crafting this dataset, we have equipped researchers and developers with robust tools that facilitate the application of both traditional time series analysis and sophisticated ML algorithms. The goal is to foster energy efficiency in campus buildings, thereby supporting the overarching objectives of cost reduction and environmental stewardship in an academic context. Through this, the University of Sharjah aims to position itself at the forefront of smart campus innovations as part of its strategic initiative towards becoming a fully integrated smart campus by 2030.

## 3 Data Description

The dataset presented is organized into four main data containers, reflecting the structure and comprehensiveness of the data collected within the university campus:

**1. Raw Data: Power Consumption Time Series**

- This segment consists of a series of tables (CSV files) that outline the power consumption data at the appliance level within the campus building. Each CSV file corresponds to a specific appliance or device, such as: File 1: Power consumption of the Coffee Machine.
- File 2: Power consumption of the Microwave.
- File 3: Power consumption of the Fridge.
- File 4: Power consumption of the Kettle.
- File 5: Power consumption of the Printer.
- File 6: Power consumption of the Desktop.
- File 7: Power consumption of the Water Dispenser.

Each file contains columns for timestamp (in YYYY-MM-DD HH:MM: SS format), starting from 2024 to 01–01 12:00:00, with power consumption (W), and additional parameters like voltage (V) and current (A).

**2. Raw Data: Environmental Conditions Time Series**

This collection comprises CSV files detailing the indoor environmental conditions captured by various sensors around the building:

- File 1: Temperature data in the Lab.
- File 2: Temperature data in the Kitchen.
- File 3: Humidity data in the Lab.
- File 4: Humidity data in the Kitchen.
- File 5: Occupancy data in the Lab.
- File 6: Occupancy data in the Kitchen/Mail Room.

Like the power consumption files, each CSV includes timestamps and respective environmental readings (e.g., °C for temperature,% for humidity).

**3. Processed Data: Power and Environmental Summary**

This set includes processed and aggregated data, offering a summarized view of power consumption and environmental conditions. This arrangement is particularly useful for quick reviews or high-level analyses. The data might be presented as additional CSV files or visual charts, depending on the processing applied. The dataset was aggregated and stored using InfluxDB, which facilitated high-resolution time series data collection. This setup enabled the precise calculation of mean environmental values, such as temperature and humidity, at predefined intervals. Such a meticulous approach ensures data integrity and granularity, which are essential for an in-depth analysis of building dynamics.

**Fig. 1:**
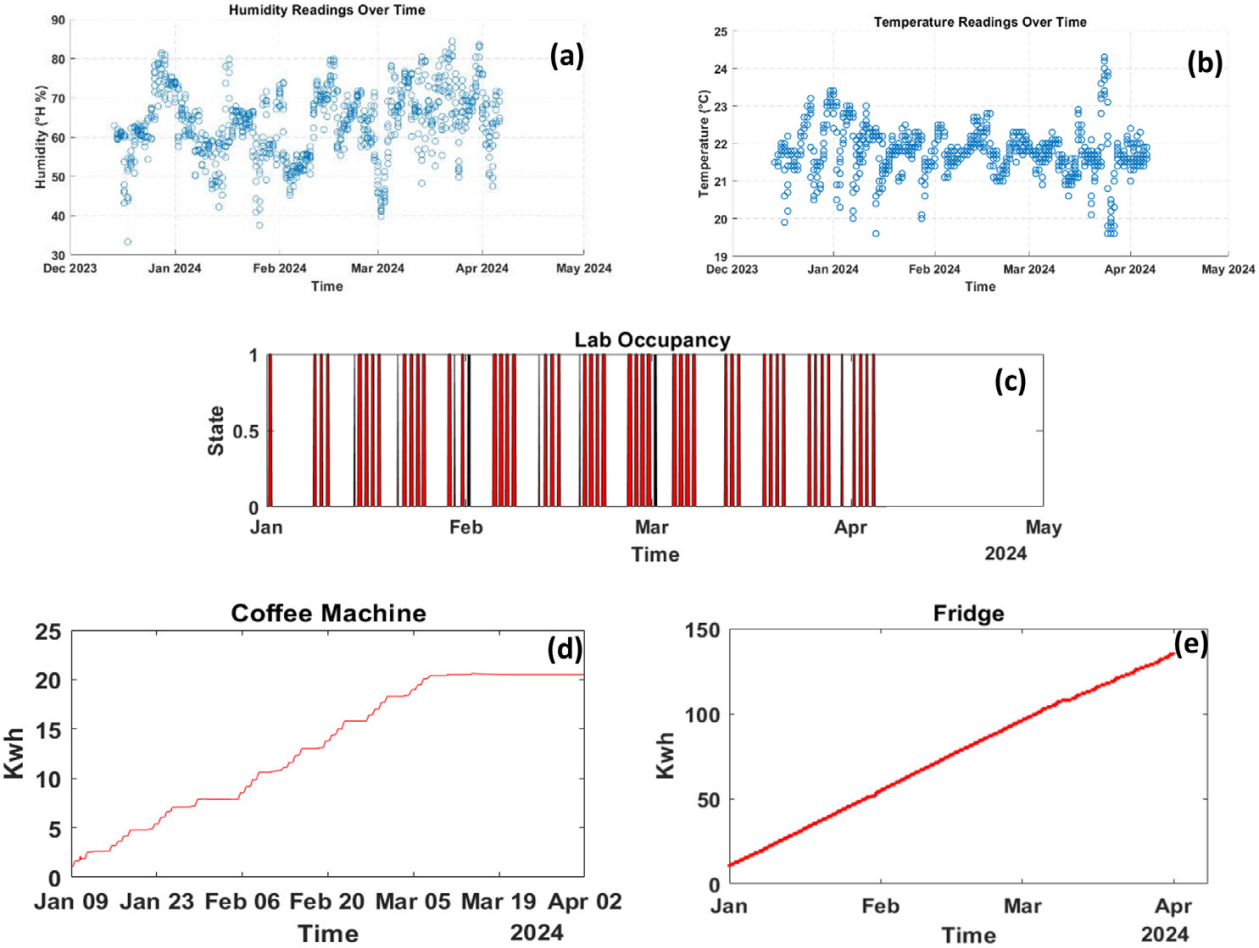
Visualization of aggregated environmental and power data in a smart building. (a) Laboratory temperature, (b) laboratory humidity, (c) occupancy levels, (d) coffee machine power usage, and (e) refrigerator power usage in the kitchen, showcasing the integration of IoT devices for enhanced monitoring and management.

Fig. 1 exemplifies the data collected within the laboratory and kitchen environments. Subfigure (a) illustrates the environmental temperature, and subfigure (b) depicts the humidity levels within the laboratory. Subfigure (c) presents the occupancy data. Subfigures (d) and (e) provide a summary of power consumption in kilowatts for the coffee machine and fridge in the kitchen, respectively. This comprehensive dataset serves as a cornerstone for analyzing the interplay between environmental conditions and energy usage, offering valuable insights into the operational efficiency and sustainability of smart building infrastructures.

**Fig. 2:**
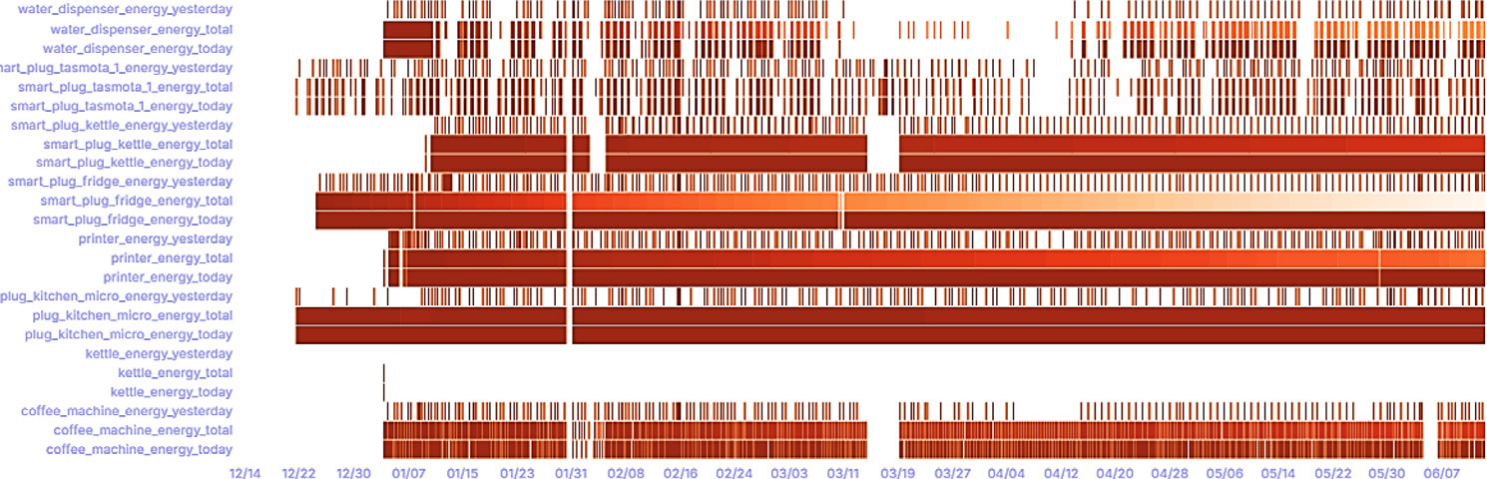
Comprehensive Energy Consumption Visualization.

Fig. 2 provides a detailed visualization of energy consumption across various devices within the smart building over an extended period. This heatmap illustrates the daily energy usage patterns of devices such as coffee machines, fridges, and desktop computers, highlighting peak usage times and potential areas for energy-saving interventions. Such visualizations aid researchers in pinpointing operational inefficiencies and developing targeted energy conservation strategies.

**Fig. 3:**
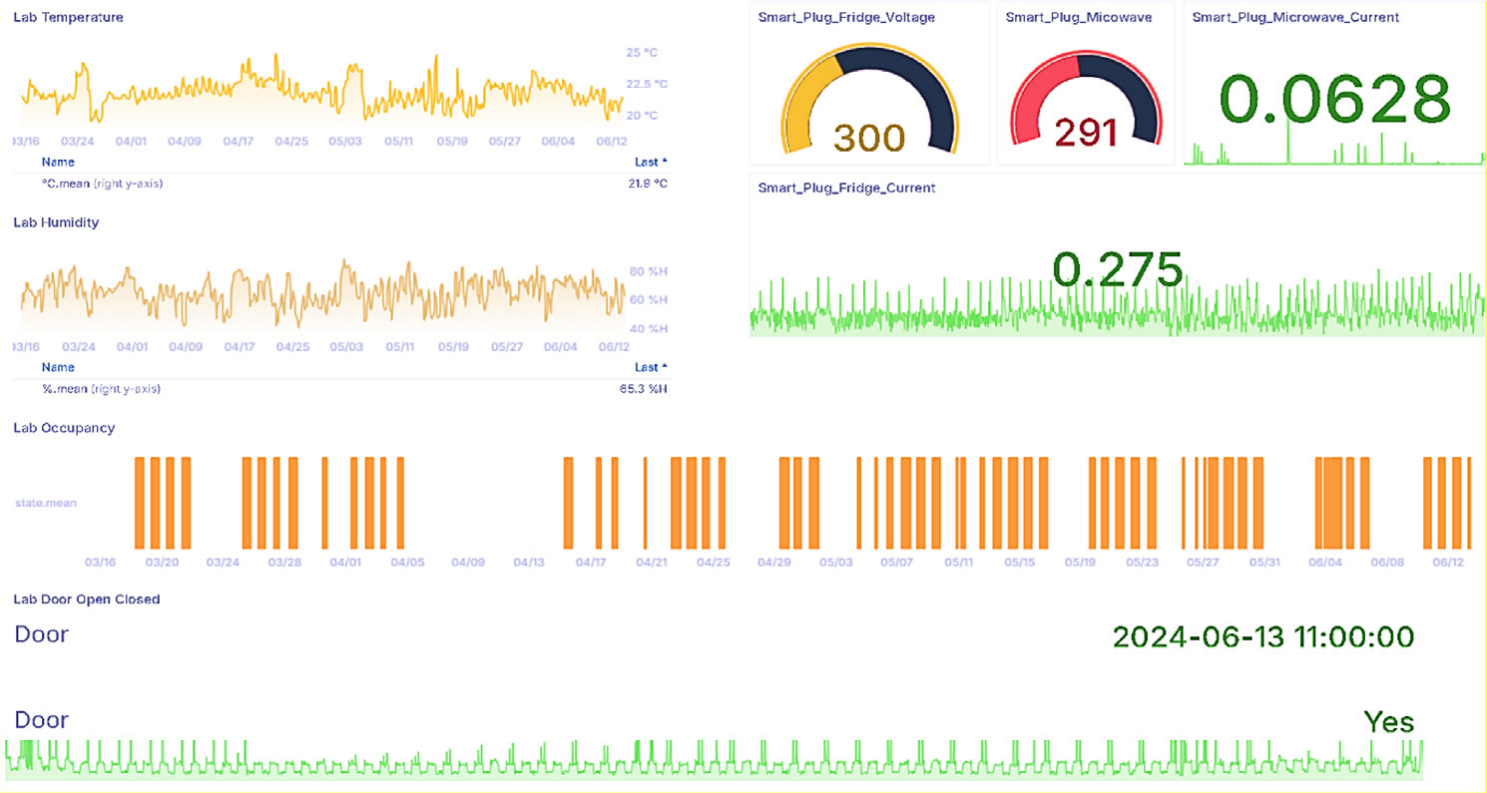
Detailed Environmental and Energy Monitoring in a Laboratory Setting.

On the other hand, Fig. 3. showcases a comprehensive set of environmental and energy consumption data from a laboratory within the smart building. It includes:

• **Lab Temperature and Humidity:** Graphs depicting the temperature and humidity levels over time, providing insights into the environmental conditions of the lab.
• **Lab Occupancy and Door Status:** A bar graph indicating occupancy levels alongside a timeline of door status, offering correlations between space usage, energy consumption, and access patterns.
• **Device-Specific Energy Metrics:** Real-time voltage and current readings from smart plugs, illustrating the detailed energy consumption of laboratory equipment. This multi-dimensional representation emphasizes the dataset's potential for supporting intricate analyses of energy and environmental dynamics within smart buildings.

**4. Documentation and Metadata**

Accompanying the raw and processed data files, documentation is provided to explain the dataset's structure, the nature of each file, and metadata detailing the sensors used, their locations, and the data collection protocols.

Each data file and folder are named and organized systematically to ensure ease of access and understandability. The CSV files allow for easy integration into various data analysis tools, enabling researchers to perform detailed analyses or apply ML techniques to uncover patterns and insights from the data. While visual aids such as graphs and charts are not included in the raw data files, they can be generated by researchers as needed to facilitate data interpretation and presentation.

## 4 Experimental Design, Materials and Methods

**Fig. 4:**
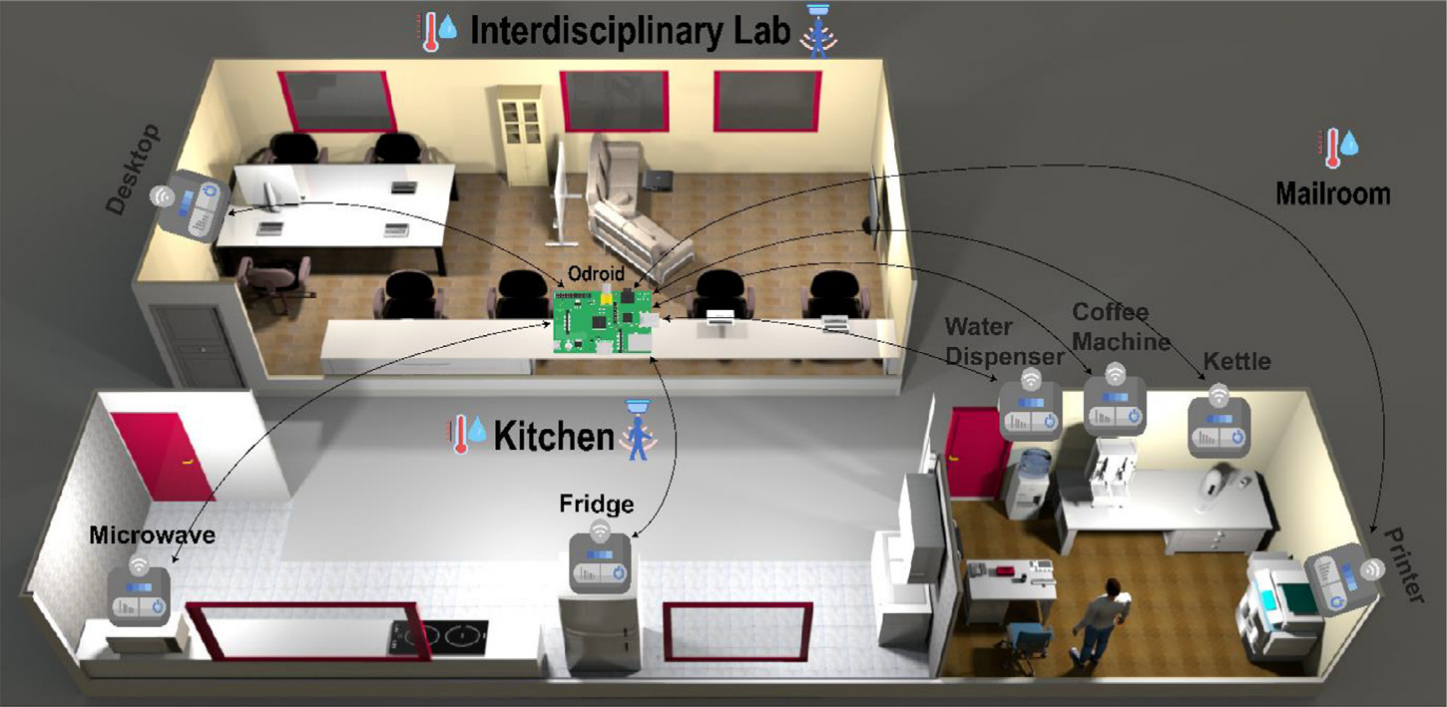
Illustration of the data collection setup in the university campus.

Our data was collected at the university campus, targeting specific areas like the Interdisciplinary Lab, Kitchen, and Mailroom as shown in Fig. 4. We commenced by setting up a central data management and edge computing hub using Odroid N^+2^ [2]. For the heart of our data collection system, we employed a robust edge computing platform capable of handling our extensive data-driven workflows.

As illustrated in Fig. 4, our setup comprised the following steps:

• **Data Collection Setup:** We employed an edge computing platform with high performance and reliability, connected to our local network infrastructure, running a custom-built smart building management system for real-time data acquisition.
• **Smart Plugs and Sensors Installation**: Smart plugs were attached to designated appliances such as microwaves, fridges, and kettles to monitor power usage.
• **Environmental Sensing**: We placed temperature, humidity, and occupancy sensors in strategic positions—like corners for maximum coverage—to gather contextual data complementing the power consumption readings. Our occupancy sensors, using PIR technology, were tested for detection range and accuracy.
• **Sensor Calibration**: We calibrated each environmental sensor against reference instruments to guarantee data accuracy.
• **Data Integrity and Connectivity Testing**: Post-installation, we conducted extensive tests to confirm the integrity of data collection and the robustness of wireless connectivity.
• **Data Acquisition:** Our system sampled data at intervals appropriate for each parameter—energy consumption data were collected every minute, and environmental conditions every five minutes.
• **Data Storage and Processing**: Initially, data was stored in a structured format using Influx-DB before being post-processed to retain only essential columns for analysis—timestamp, appliance identifier, power consumption, and environmental conditions.
• **Database Management**: Given the high volume of data collected, we implemented database management strategies to handle, compress, and export the data efficiently while maintaining data integrity.

### 4.1 Utilization of Markov transition fields (MTF) for time series analysis of energy consumption

The analysis of energy consumption patterns across various appliances, from coffee machines to water dispensers, requires a method that not only captures the inherent temporal dynamics but also facilitates a comprehensive and intuitive interpretation. The Markov Transition Field (MTF) method [5], applied to time series data, offers a distinctive approach by transforming one-dimensional time series into two-dimensional matrices, enabling the application of image analysis techniques to time series data.

This transformation is particularly beneficial for visualizing complex patterns in energy consumption, identifying cyclical behaviors, and detecting anomalies. By converting the time series into a matrix form, MTF encapsulates time-dependent relationships within the data, providing a richer context for analysis compared to traditional time series analysis methods.

The MTF stands out in visualizing time series data by transforming it into 2D images, enabling the use of image processing techniques for deeper analysis. Unlike traditional plots, MTF reveals patterns and relationships not immediately apparent, offering a unique perspective on data dynamics. This makes MTF particularly effective for comparing multiple time series and uncovering insights in fields like energy consumption, where detailed analysis can drive informed decisions.

### 4.2 Mathematical formulation of the MTF method

The core idea of the MTF method is to represent the time series as a two-dimensional image, facilitating the application of various image processing techniques. The transformation process involves several mathematical steps:

• **Normalization**: Initially, the time series data $x_{i}$ is normalized to ensure all values are within the range [0, 1]. This normalization is crucial for maintaining consistency in the transformation process:
  (1) $$x_{i}^{\prime }=\frac{x_{i}-\min\left(x\right)}{\max\left(x\right)-\min\left(x\right)}\;$$

where $x_{i}^{{}^{\prime }}$ is the normalized value at time.

• **Angular Transformation**: The normalized values are then converted into angular values using the arccosine function, mapping each data point onto a unit circle:
  (2) $$\emptyset x_{i}=arccas\left(x_{i}^{\prime }\right)$$

This angular transformation provides a unique perspective on the data, emphasizing the cyclical patterns and relationships within the time series.

**MTF Matrix Construction:** The MTF matrix M is constructed by calculating the cosine of the sum of angles between each pair of time points:

(3) $$M_{i,j}=\cos\emptyset \left(x_{i}\right)+\cos\emptyset \left(x_{j}\right)$$

Here $M_{i,j}$ represents the angular relationship between time points iandj encapsulating the temporal dynamics in a matrix form.

• Quantile Bins and Windowing:
  ○ **Quantile Bins (q):** The time series is divided into q quantile bins, which categorize the data points based on their distribution, aiding in the analysis of state transitions within the time series.
  ○ **Window (w):** The window size w determines the granularity of the MTF matrix. A larger window captures more detailed information but results in a larger matrix, while a smaller window offers a more abstract representation.

### 4.3 Application in energy consumption analysis

In the context of energy consumption analysis, the MTF method allows for the examination of patterns and correlations across different appliances over time. By transforming the energy consumption data into a matrix form, we can visually inspect the data for patterns, compare the consumption behavior of different appliances, and apply advanced image processing techniques for further analysis.

This approach is particularly advantageous for detecting cyclical usage patterns, identifying peak consumption times, and understanding the temporal relationships in energy usage, which are critical for optimizing energy consumption and enhancing efficiency in energy management systems.

The MTF method provides a robust framework for transforming energy consumption time series into a more analyzable and visually interpretable form, offering deeper insights into the dynamics of energy usage patterns.

### 4.4 Occupancy and environmental data

• **Occupancy in Lab**: Similarly, the lab's motion MTF plot can indicate occupancy trends, offering a context for the energy usage of lab equipment
• **Occupancy in Kitchen**: The MTF plot for motion data in the kitchen can reveal occupancy patterns, which can be correlated with energy consumption in kitchen appliances.

**Fig. 5:**
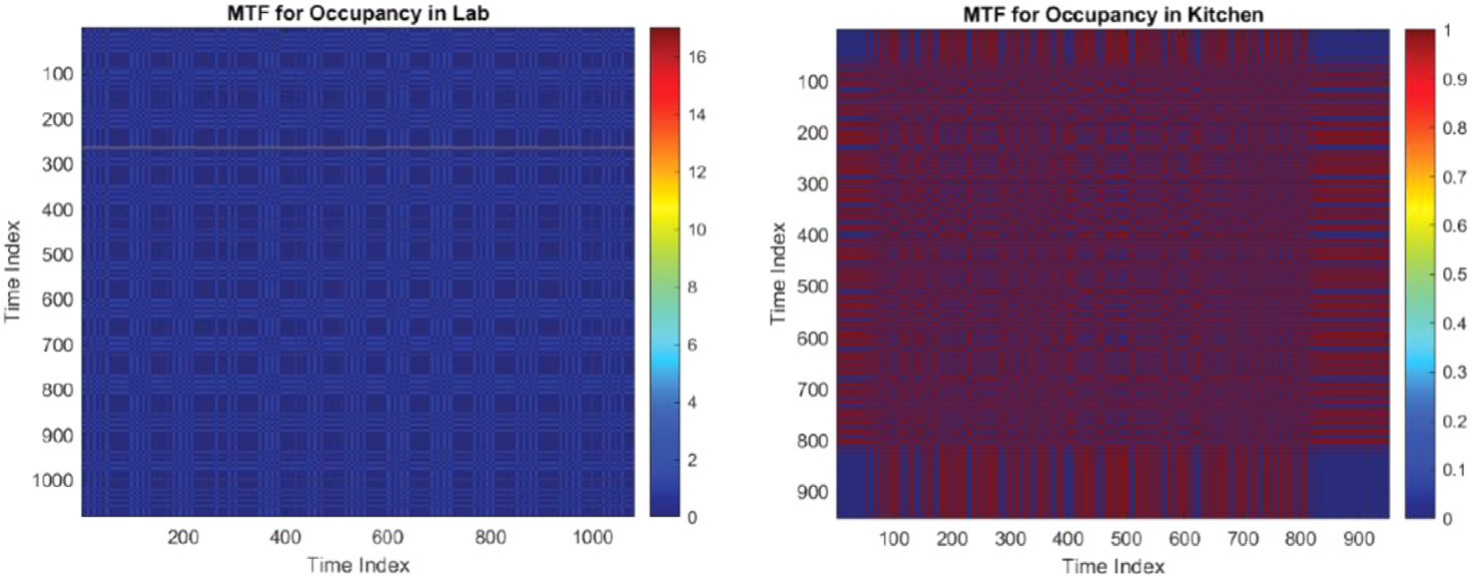
Comparative MTF heatmaps depicting occupancy trends; on the left, the lab exhibits minimal variability over time, while on the right, the kitchen shows dynamic occupancy levels.

In Fig. 5, we observe two Matrix Time-Function (MTF) heatmaps: the left panel (Fig. 5 left) showcases the occupancy in the lab, with a consistent low-activity pattern indicated by a predominant deep blue color; conversely, the right panel (Fig. 5 right) displays occupancy in the kitchen, characterized by intermittent high-activity periods as denoted by the fluctuating presence of warmer colors in the heatmap.

• **Temperature in Kitchen and Mailroom**: On the other hand, the kitchen and mailroom temperature MTF plot might show how ambient temperature varies and interacts with the usage patterns of kitchen and mailroom appliances.
• **Temperature in Lab**: For the lab, the MTF visualization of temperature can provide insights into environmental control systems and their energy implications.

**Fig. 6:**
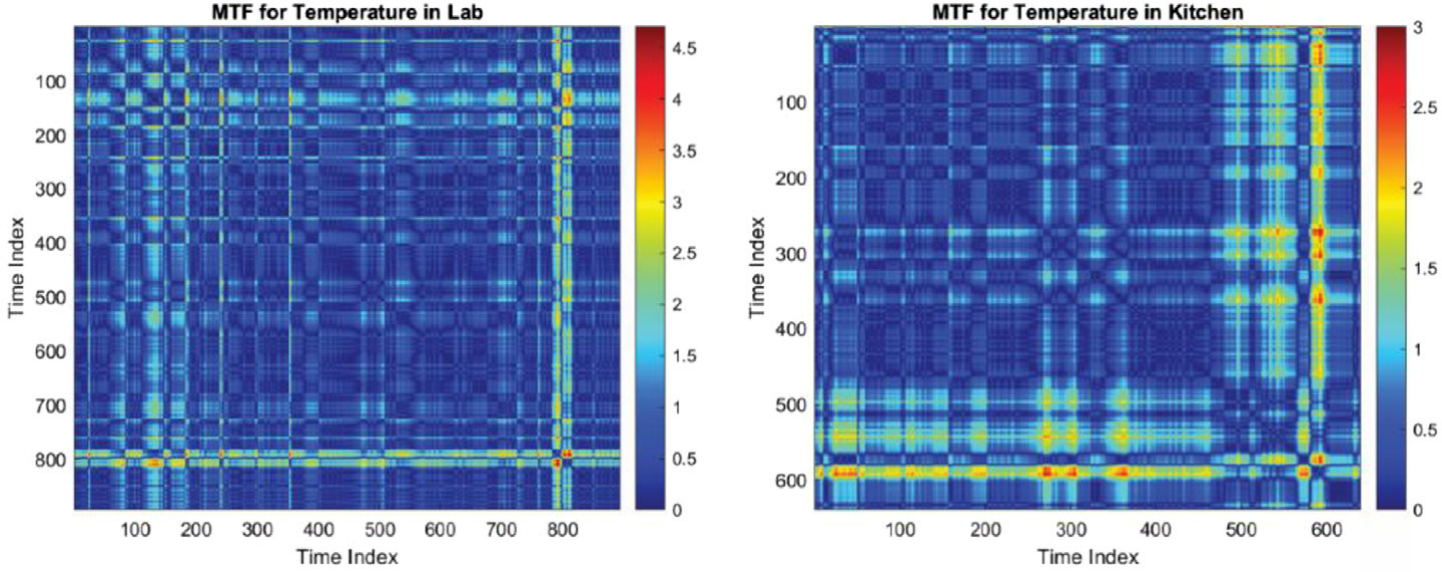
MTF heatmaps illustrate the temporal temperature fluctuations in a lab (left) and a kitchen (right), with sporadic peaks in the lab and more frequent high-temperature events in the kitchen.

In these Matrix Time-Function (MTF) heatmaps labeled as Fig. 6, the left panel displays the temperature variation within a laboratory setting over a time index. Occasional hotspots suggest intermittent temperature peaks. The right panel shows the temperature dynamics within a kitchen/mailroom environment, where frequent and pronounced spikes in temperature are evident, indicating regular activity likely associated with cooking or appliance use.

• **Humidity in the Kitchen and Mailroom**: MTF plots for humidity data can further contextualize the energy usage patterns, especially for equipment sensitive to environmental conditions.

**Fig. 7:**
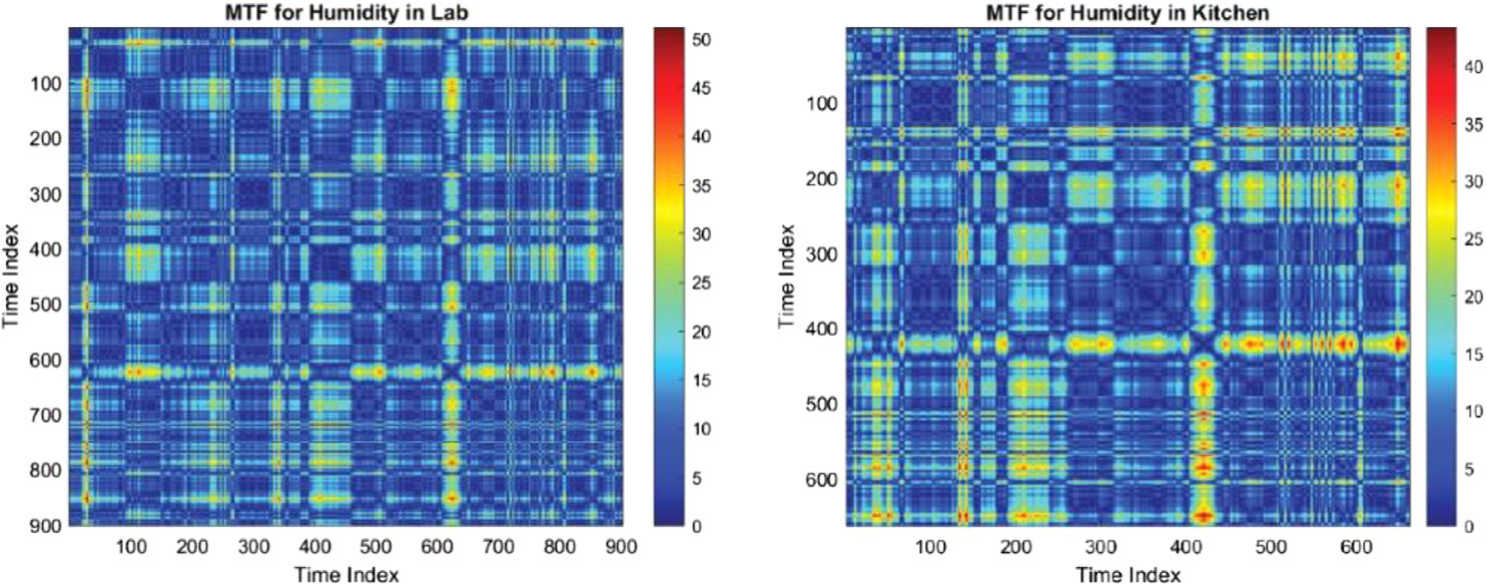
MTF heatmaps for humidity levels, with the lab (left) showing occasional concentration and the kitchen (right) exhibiting frequent high-humidity events.

These MTF heatmaps in Fig. 7 capture humidity patterns, where the lab demonstrates isolated instances of increased humidity, likely due to specific experiments or activities. In contrast, the kitchen presents regular and pronounced high-humidity intervals, possibly from cooking or the use of appliances that generate steam.

### 4.5 MTF visualizations for energy consumption

The application of the MTF methodology on energy consumption data from various appliances yields insightful visual representations that facilitate a deeper understanding of energy usage patterns. Below, we describe the figures obtained from the MTF transformation of each piece of equipment processed and validated, which would then be ready for a multitude of analyses, such as predictive maintenance, energy efficiency optimization, and in-depth behavioral studies on energy consumption patterns across the campus.

• **Coffee Machine**: The MTF plot for the coffee machine reveals distinct patterns indicative of its usage cycles. The matrix might show concentrated areas where energy consumption is heightened, corresponding to peak usage times.
• **Desktop**: For the desktop, the MTF visualization can illuminate its operational patterns, highlighting periods of continuous use or standby modes.
• **Fridge**: The MTF plot for the fridge is expected to display periodic cycles reflecting its cooling mechanisms and door-opening events.
• **Microwave**: The MTF plot for the microwave could reveal usage spikes and correlate them with specific times, highlighting its intermittent usage.
• **Printer**: The printer's MTF plot might exhibit patterns related to office hours and print job occurrences.
• **Water Dispenser**: For the water dispenser, the MTF plot can provide insights into daily usage patterns and potential standby energy consumption.
• **Kettle**: The kettle's MTF visualization is likely to show sporadic usage patterns, emphasizing boiling events.
• **General**: Analyzing a general energy consumption profile, the MTF plot can indicate overall energy usage trends and anomalies over time.

**Fig. 8:**
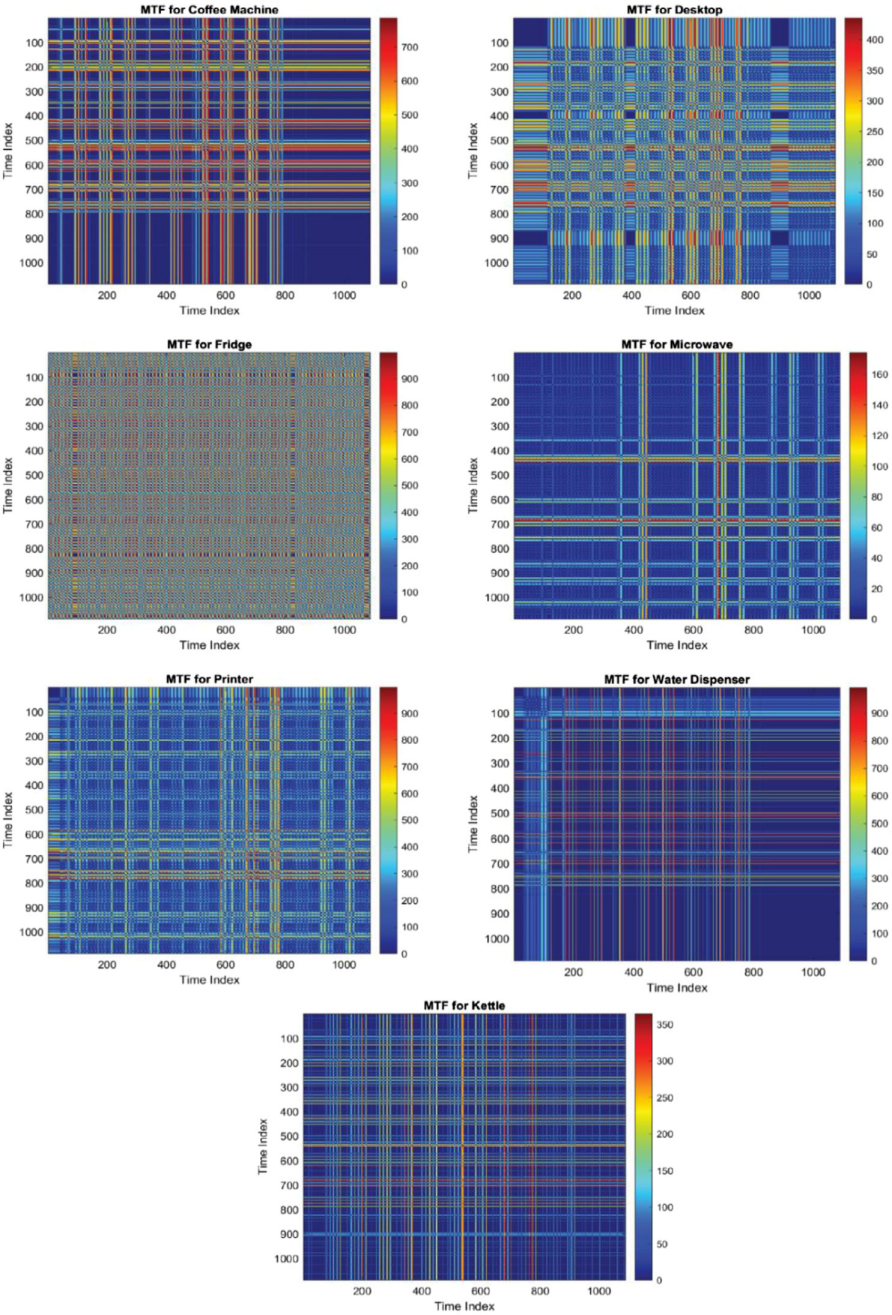
Set of MTF heatmaps representing energy consumption for various office appliances, revealing distinct usage patterns for coffee machines, desktops, fridges, microwaves, printers, water dispensers, and kettles across a standardized time index.

To sum up, the MTF image visualizes the relationship between different time points in the energy consumption data of the above appliances from January 1st, 2024, to June 20st, 2024. Each axis represents time steps in the normalized time series, where the data has been transformed to show how energy usage patterns evolve over time. Brighter areas in the image indicate periods with similar energy consumption levels, while darker areas signify times with distinct energy usage patterns. This visualization helps in identifying patterns and trends in the energy data that may not be immediately apparent in the raw time series. Fig. 8, presents examples of the MTF visualization for different appliances, demonstrating how this method can be applied to analyze and compare energy consumption patterns across various devices. The MTF images offer a unique perspective on the temporal dynamics of energy usage, providing a powerful tool for identifying patterns, similarities, and anomalies in the data over time.

**Fig. 9:**
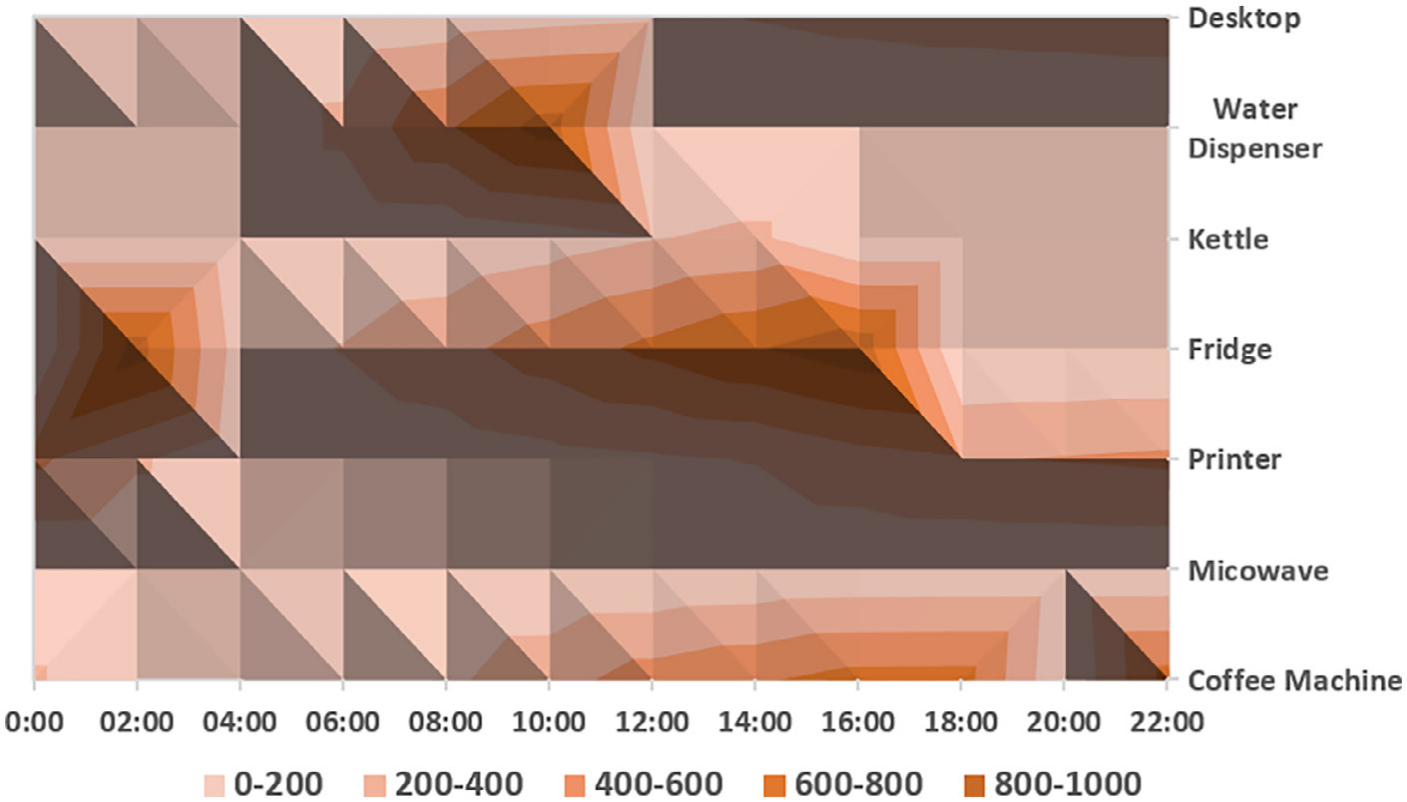
Stacked area chart displaying daily office appliance usage by time, with color-coded layers indicative of varying consumption levels. (For interpretation of the references to color in this figure legend, the reader is referred to the web version of this article.)

In Fig. 9, we observe a time series stacked area chart delineating the power consumption patterns of various appliances over a 24-hour period, with the x-axis quantifying time from 0:00 to 24:00 h. The y-axis, while not explicitly labeled, is inferred to measure power usage in watts (W). The stratification of colors represents different appliances—namely desktop computers, water dispensers, kettles, refrigerators, printers, microwaves, and coffee machines. Each layer's thickness correlates with the device power consumption at given times, categorized into wattage brackets as delineated by the legend (0–200 W, 200–400 W, etc.). The visual data suggest temporal variation in usage, with certain devices like coffee machines showing increased consumption during early hours, and desktop computers exhibiting peak usage during conventional office hours.

## Limitations

One limitation of the dataset is its short duration, spanning only 5 to 6 months. This temporal scope may restrict the ability to capture long-term trends and seasonal variations in energy usage and environmental conditions, which are critical for comprehensive energy management and sustainability studies. Furthermore, while the data provides insights into campus energy dynamics, its specificity to the university environment may not generalize to other types of buildings or different climatic conditions. Additionally, the size of the dataset, although substantial, may not be adequate for training more complex deep learning models without employing data augmentation techniques. This could affect the development of robust predictive models for energy consumption. Finally, despite careful calibration, there is an inherent limitation in sensor accuracy, which could introduce slight measurement errors. However, this is a common challenge in IoT-based data collection and is mitigated through regular calibration and validation against reference instruments (Table 1).

**Table 1:** Specifications of appliances monitored [3].

#	Category	Device Name	Manufacturer	Location	Connectivity	Compatibility	Features
**1 Fridge**	Smart Plugs	Local Bytes Power	Local Bytes	Kitchen	Wi-Fi	Tasmota/ESP Home	Real-time energy monitoring
**2 Microwave**							
**3 Desktop**				Lab			
**4 Water Dispenser**				Mailroom			
**5 Kettle**							
**6 Coffee machine**							
**7 Printer**							
**8 Weather forecasting**	Temperature Sensors	Sonoff SNZB-02	Sonoff	Various	Zigbee	eWeLink, Alexa	Temperature & Humidity monitoring
**9 Occupancy**	Motion Sensors	Sonoff SNZB-03		Various		eWeLink, IFTTT	Motion detection, Instant alert notification

## Ethics Statement

This data was collected in accordance the Declaration of Helsinki and have obtained ethical approval from the Faculty of Computer Science Department, at University of Sharjah (UoS). It is important to note that the collected data focuses solely on energy consumption and environmental conditions, ensuring no personal data related to the users of the rooms or devices were gathered, highlighting our commitment to user privacy and ethical standards.

## CRediT authorship contribution statement

**Adel Oulefki:** Methodology, Writing – original draft, Writing – review & editing. **Abbes Amira:** Conceptualization, Methodology, Writing – review & editing, Validation, Supervision. **Fatih Kurugollu:** Writing – review & editing, Validation, Supervision. **Bassel Soudan:** Writing – review & editing, Validation, Supervision.

## Data Availability

Dataset-of-IoT-Based-Energy-and-Environmental-Parameters-in-a-Smart-Building-Infrastructure (Original data) (ANDS).
